# Association of body mass index and dietary intake with mild cognitive impairment and dementia: a retrospective cohort study

**DOI:** 10.1186/s12877-022-03700-5

**Published:** 2023-01-03

**Authors:** Apisit Manacharoen, Kulapong Jayanama, Sirasa Ruangritchankul, Prin Vathesatogkit, Piyamitr Sritara, Daruneewan Warodomwichit

**Affiliations:** 1 Department of Medicine, Faculty of Medicine Ramathibodi Hospital, Mahidol University, Bangkok, Thailand; 2 Chakri Naruebodindra Medical Institute, Faculty of Medicine Ramathibodi Hospital, Mahidol University, Samut Prakan, Thailand; 3 Division of Geriatrics Medicine, Department of Medicine, Faculty of Medicine Ramathibodi Hospital, Mahidol University, Bangkok, Thailand; 4 Division of Cardiology, Department of Medicine, Faculty of Medicine Ramathibodi Hospital, Mahidol University, Bangkok, Thailand; 5 Division of Nutrition and Biochemical Medicine, Department of Medicine, Faculty of Medicine Ramathibodi Hospital, Mahidol University, Bangkok, Thailand

**Keywords:** Body mass index, Nutrition, Dietary intake, Mild cognitive impairment, Dementia

## Abstract

**Background:**

The prevalence of cognitive impairment in older adults is gradually increasing, and this is leading to many adverse outcomes. Common causes of cognitive impairment in advancing age are mild cognitive impairment (MCI) and dementia. However, how the nutritional status and nutrient intake are related to MCI and dementia is controversial. Therefore, we aimed to evaluate the association of body mass index (BMI) and dietary intake with the risk of MCI and dementia.

**Methods:**

This retrospective cohort study involved 821 participants aged ≥ 50 years from a previous population-based cohort study: the Electricity Generating Authority of Thailand (EGAT) study in 2013–2014 (baseline) and 2018–2019 (follow-up). Dietary intake was recorded using a 12-month self-reported food frequency questionnaire. MCI and dementia were diagnosed according to the Diagnostic and Statistical Manual of Mental Disorders (DSM-5) criteria using the Montreal Cognitive Assessment with ADL and the Kessler Psychological Distress Scale (K10) at study entry and at the 5-year follow-up.

**Results:**

Among the 821 participants, the mean age was 60.0 ± 4.3 years, and the incidence rate of MCI and dementia was 42.5 and 11.2 per 1,000 person-years, respectively. The rate of MCI and dementia was higher in participants aged ≥ 60 years and with an education level of < 7 years. The rate of MCI was also higher in those with a BMI of ≥ 25 kg/m^2^ and type 2 diabetes. Compared to BMI 18.5–22.9 kg/m^2^, BMI of ≥ 25 kg/m^2^ (odds ratio 1.91 [95% confidence interval, 1.12–3.26], *p* < 0.001) was associated with an increased risk of MCI after adjusted for age, education level, and type 2 diabetes. Regarding dietary intake, fresh red meat consumption was inversely associated with the risk of MCI (*p* = 0.037) and dementia (*p* = 0.011) after adjusting for age, education level, type 2 diabetes, and BMI.

**Conclusion:**

Obesity was associated with a greater risk of MCI. Moreover, low consumption of fresh red meat could be a risk factor for MCI and dementia. Further studies are required to confirm and explain these findings.

**Supplementary Information:**

The online version contains supplementary material available at 10.1186/s12877-022-03700-5.

## Background

By 2050, the prevalence of MCI and dementia is predicted to reach 106.8 and 131.5 million people worldwide, respectively [1]. The percentage of people who progress from MCI to dementia, especially Alzheimer’s disease, is 10–15% at one year and 32–38% at five years. Mild cognitive impairment (MCI) is a transitional state of cognitive decline in at least one area of neuropsychological function. It represents a stage between normal cognitive function and dementia [2] and is characterized by preserved activities of daily living (ADL). While dementia, as a chronic or progressive disease of brain deterioration, consists of impairment of several cognitive domains, including memory, thinking, comprehension, calculation, learning, language, and judgment [3]. Epidemiological studies of older people have shown that the mortality rate in patients with cognitive impairment also increased depending on the etiology, e.g., cerebral ischemia, trauma, metabolic disturbance, or psychiatric illness [4–7]. MCI and dementia are public health concerns and significant socioeconomic burdens in aging societies.

. Many risk factors for cognitive decline have been identified, including chronic diseases such as hypertension, diabetes mellitus, hyperlipidemia, and depression, as well as adverse conditions such as current smoking and sleep disturbance [8]. In contrast, high formal education levels, physical activity, social engagement, and an appropriate nutritional status are protective factors against cognitive decline [9, 10]. Inflammatory processes, that resulted in neuronal damage of ascending cholinergic neurons and large pyramidal cells in the cerebral cortex, were also an etiology of cognitive impairment [11].

Malnutrition, including both undernutrition and overnutrition, is a common health problem in older people. Previous studies have revealed an association of being underweight with poor quality of life, reduced functional abilities and increased mortality [12, 13]. Moreover, malnourished patients have a higher risk of both MCI and dementia [14–16]. Adiposity can also increase metabolic risk and may cause cerebrovascular diseases and neurodegeneration [17]. A previous study [18] showed the rate of MCI increased in older women with low body mass index (BMI) and older men with high BMI. Not only the patient’s overall nutritional status but also the details of his or her dietary intake should be areas of focus in clinical assessments. With respect to the relationship between individual nutrients and cognition, recent studies have revealed protective effects between cognitive decline and specific nutrients, such as vitamin B (B6, B12, and folate), antioxidants (carotenoids, vitamin C, vitamin E, selenium, flavonoids, and polyphenols), vitamin D, monounsaturated fatty acids, and omega-3 fatty acids [19–21]. However, the results were inconsistent and clinically non-significant.

Because many nutrients are consumed each day, the whole food and dietary pattern should be of greater concern and may more strongly affect overall health than individual nutrients. Previous observational studies have shown that healthy dietary patterns, including the Mediterranean diet, Dietary Approaches to Stop Hypertension (DASH), healthy Nordic diet, and Japanese diet, can slow cognitive decline as well as decrease adverse health outcomes [16, 22–24]. Concerning dietary groups, reduced intake of meat, meat products, and sugary drinks may raise cognitive performance. Previous studies from the UK biobank [25, 26] revealed that coffee and tea drinking was associated with a lower risk of stroke and dementia, but processed meat intake was a potential risk factor for dementia. Nonetheless, the evidence of a relationship between the types or amount of meat intake and cognitive performance is limited.

How the nutritional status and dietary consumption affect cognitive impairment, especially MCI, remains inconclusive. This study aimed to evaluate the association of BMI with MCI and dementia risk and to explore the relationship of dietary intake with MCI and dementia risk.

## Methods

### Study population and design

**Fig. 1:**
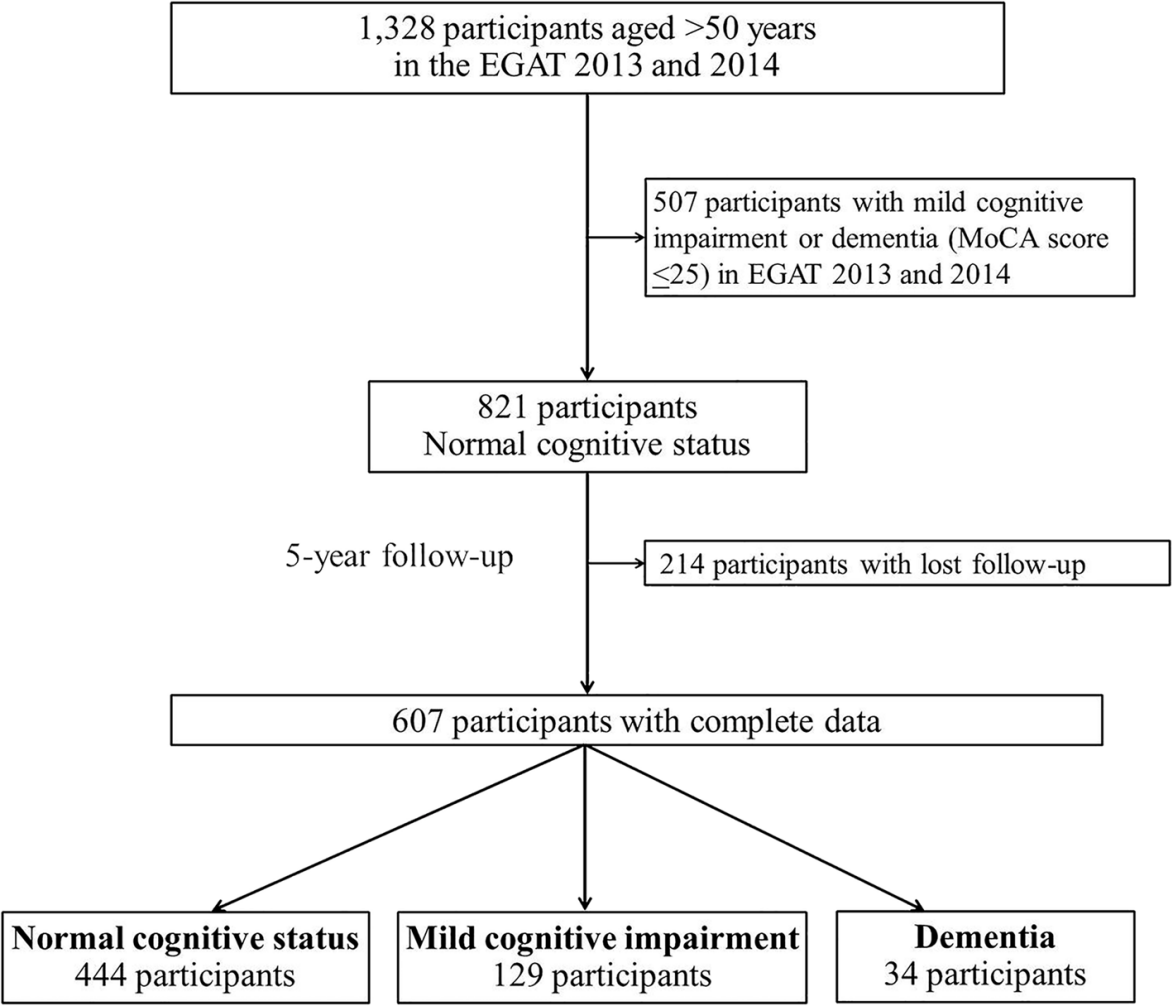
Participant flow chart. EGAT, Electricity Generating Authority of Thailand study; MoCA, Montreal Cognitive Assessment

This retrospective cohort study initially included 1,328 participants aged ≥ 50 years from the Electricity Generating Authority of Thailand (EGAT) study in the years 2013 (EGAT2) and 2014 (EGAT3). The EGAT study was a population-based cohort study that enrolled EGAT employees aged ≥ 35 years from more than 30 occupations and followed up every five years [27]. At baseline, demographic data (age, sex, education level, smoking, and alcoholic drinking), health conditions (chronic diseases), prescribed medications, the Barthel index (BI) scores, the Lawton instrumental activities of daily living (L-IADL) scores, the 10-item Kessler Psychological Distress Scale (K10) scores, and the Montreal Cognitive Assessment (MoCA) scores were collected under face-to-face interviews. Physical examinations, including body weight, height, and waist and hip circumference, were performed by well-trained personnel. Barthel Index (BI), Lawton instrumental activities of daily living, MoCA scores, and K10 scores were re-evaluated at the 5-year follow-up. At baseline, we excluded 507 participants previously diagnosed with MCI or dementia. We also excluded 214 participants who lost to follow-up. Finally, 607 participants, aged ≥ 50 years and having normal cognitive status, were included in the study. Participants were categorized into three groups by cognitive status at 5-year follow-up: normal cognition, MCI, and dementia **(**Fig. 1**)**.

Written informed consent was obtained from all participants and their legal guardian. The protocol was approved by the Institutional Review Board of the Faculty of Medicine Ramathibodi Hospital, Mahidol University (approval number COA. MURA2020/1450).

### Definitions

#### Cognitive status

The cognitive status was assessed in all participants aged ≥ 50 years using the Thai version of the MoCA. The MoCA is a well-calibrated and widely used assessment tool to evaluate cognition in eight domains: visuospatial/executive function, naming, memory, attention, language, abstraction, delayed recall, and orientation [28, 29]. The MoCA score ranges between 0 and 30, where a score of ≤ 25 indicates impairment in cognitive performance. The capacity for independence in everyday activities of all participants was evaluated using BI and L-IADL in the Thai version [30–33]. The BI is composed of ten variables describing ADL and mobility. The BI score ranges between 0 and 20, where a score of < 12 indicates impaired ADL. The L-IADL evaluates eight domains of instrumental activities of daily living (IADL). The L-IADL score ranges between 0 and 8, where a score of < 8 indicates increased dependence of IADL. Psychological stress or mental disorders of participants were evaluated using the K10 score [34]. The K10 is a validated tool, designed to assess nonspecific psychological distress and mental disorders. The K10 consists of 10 items on a 5-point scale with total scores ranging between 10 and 50, where a score of < 20 is indicative of normal mental status.

A normal cognitive status was defined as normal cognitive performance (MoCA > 25), ADL (BI ≥ 12), and IADL (L-IADL = 8). We defined MCI and dementia using face-to-face clinical diagnosis following the Diagnostic and Statistical Manual of Mental Disorders (DSM-5) criteria [35]. MCI (minor neurocognitive disorders) was defined as a decline in cognitive function without an impaired capacity for independence in everyday activities, which was not interfered with by mental disorders (K10 < 20) or delirium. Dementia (major neurocognitive disorder) was defined as both cognitive impairment and a decline in capacity for independence in everyday activities.

#### Body mass index (BMI)

The BMI was calculated as weight in kilograms divided by the square of height in meters. The World Health Organization recommendations for Asian populations were used to categorize individuals into four BMI groups: <18.5 kg/m^2^ (underweight), 18.5–22.9 kg/m^2^ (normal weight), 23.0–24.9 kg/m^2^ (overweight), and ≥ 25 kg/m^2^ (obese) [36]. We used a normal weight as a reference BMI.

#### Dietary data

Dietary consumption was assessed using a self-reported semi-quantitative food frequency questionnaire (FFQ) [37, 38] which included 40-food items. The validated FFQ was translated into Thai. Participants were asked to recall their average frequency of dietary consumption in standard serving size during the past 12 months after the well-trained personnel demonstrated the portion size of each food to participants. We calculated daily dietary intake by multiplying the average number of daily servings by assigned portion sizes. We constructed and analyzed nine major diet groups based on the interest and potential association with study outcomes: fresh red meat, processed meat, white meat (poultry, fish, and seafood), animal protein (fresh red meat, processed meat, white meat, and meat organs), fruits, vegetables, drinks with added sugar, refined carbohydrates, and unrefined carbohydrates. Each dietary intake group was divided into three groups by tertiles of participants, and the median (minimum – maximum) dietary intake in each tertile was presented in Additional file 1: Table S1. We used the tertile 1 of each dietary intake group as a reference.

### Statistical analyses

Statistical analyses were conducted using IBM SPSS Statistics for Windows, Version 24.0 (IBM Corp., Armonk, NY, USA). Categorical variables were compared using the chi-square test to determine the differences between groups and are reported as N (%). The mean difference of continuous variables was compared using ANOVA with the Bonferroni post hoc test and is reported as mean ± standard deviation (SD).

Regarding objective 1 (to evaluate the association of BMI with MCI and dementia risk), we evaluated the association of BMI with MCI and dementia using univariate and multivariate multinomial logistic regression analyses. All potential confounding factors (*p*-value of < 0.1 in the univariate regression analyses **(**Additional file 1: Table S2)) were considered as potential covariates, including age (< 60 and ≥ 60 years), education level (< 7 and ≥ 7 years), and type 2 diabetes [8, 10]. The multivariate multinomial logistic regression model was adjusted for these potential covariates.

Regarding objective 2 (to explore the relationship of dietary intake with MCI and dementia risk), we also evaluated the association of dietary intake with MCI and dementia using univariate and multivariate multinomial logistic regression analyses. All multivariate multinomial logistic regression models were also adjusted for potential covariates, including age, education level, type 2 diabetes, and BMI (< 18.5 kg/m^2^, 18.5–22.9, 23.0–24.9, and ≥ 25). A *p*-value of < 0.05 was considered statistically significant using a two-tailed test for independent samples.

## Results

### Baseline characteristics of study participants

At baseline, 821 participants with normal cognitive status were included in this study. However, 214 participants lost to follow-up. Then, 607 participants with complete follow-up data were analyzed **(**Fig. 1**)**. There was no statistically significant difference in baseline characteristics of participants between the analyzed and lost follow-up (Additional file 1: Table S3). Among 607 participants, their mean age was 55.9 ± 4.4 years, and 18.5% of the total population was aged ≥ 60 years. The majority of participants were male (69.4%). The percentage of participants underweight, normal weight, overweight, and obese were 2.5, 28.2, 23.4, and 46.0, respectively. The three most frequent comorbidities were dyslipidemia (54.4%), hypertension (54.2%), and type-2 diabetes (14.2%). At the 5-year follow-up, 21.3% had developed MCI, and 5.6% had developed dementia by five years **(**Table 1**)**. The incidence rates of MCI and dementia were 42.5 and 11.2 per 1,000 person-years, respectively.

**Table 1:** Baseline characteristics of participants stratified by cognitive status at 5-year follow-up (normal cognition, MCI, and dementia)

**Baseline characteristics**	**Total** ***N*** ** = 607**	**Cognitive status at 5-year follow-up**					
		**Normal**	**MCI**	***p*** **-value**	**Dementia**	***p*** **-value**	***p*** **-value**
		***N*** **= 444 (73.1%)**	***N*** **= 129 (21.3%)**	**Normal VS. MCI**	***N*** ** = 34 (5.6%)**	**Normal VS. Dementia**	
Age, years	55.9 ± 4.4	55.4 ± 4.1	56.6 ± 4.8	**0.026**	58.6 ± 5.0	**<0.001**	**<0.001**
Age of ≥60 years	112 (18.5)	66 (14.9)	30 (23.3)	**0.025**	16 (47.1)	**<0.001**	**<0.001**
Sex, male	421 (69.4)	303 (68.2)	97 (75.2)	0.130	21 (61.8)	0.436	0.197
Body mass index, kg/m^2^	25.0 ± 3.9	24.8 ± 3.8	25.8 ± 3.9	**0.023**	24.7 ± 4.4	1.000	**0.026**
Body mass index, kg/m^2^				**0.046**		0.610	0.130
<18.5	15 (2.5)	10 (2.3)	3 (2.3)		2 (5.9)		
18.5–22.9	171 (28.2)	136 (30.6)	25 (19.4)		10 (29.4)		
23.0–24.9	142 (23.4)	106 (23.9)	29 (22.5)		7 (20.6)		
≥25.0	279 (46.0)	192 (43.2)	72 (55.8)		15 (44.1)		
Education level of <7 years	139 (22.9)	73 (16.4)	51 (39.5)	**<0.001**	15 (44.1)	**<0.001**	**<0.001**
Underlying diseases							
Dyslipidemia	330 (54.4)	248 (55.9)	64 (49.6)	0.229	18 (52.9)	0.742	0.449
Hypertension	329 (54.2)	234 (52.7)	75 (58.1)	0.316	20 (58.8)	0.491	0.472
Type 2 diabetes	86 (14.2)	55 (12.4)	26 (20.2)	**0.026**	5 (14.7)	0.694	0.083
Ischemic heart disease	18 (3.0)	12 (2.7)	4 (3.1)	0.809	2 (5.9)	0.289	0.571
Thyroid disorder	16 (2.6)	13 (2.9)	1 (0.8)	0.163	2 (5.9)	0.341	0.194
Stroke	6 (1.0)	3 (0.7)	3 (2.3)	0.105	0 (0.0)	0.631	0.208
Smoking	226 (37.2)	160 (36.0)	56 (43.4)	0.148	10 (29.4)	0.437	0.195
Alcohol drinking	454 (74.8)	334 (75.2)	96 (74.4)	0.908	24 (70.6)	0.548	0.830

*MCI* mild cognitive impairment

Data are presented as mean ± standard deviation or n (%).

Boldface *p*-values are statistically significant.

Compared with participants who had normal cognition, the number of participants with MCI and dementia was significantly higher among those aged ≥ 60 years (23.3% vs. 14.9%, *p* = 0.025 and 47.1% vs. 14.9%, *p* < 0.001) and those with low education levels (< 7 years) (39.5% vs. 16.4%, *p* < 0.001 and 44.1% vs. 16.4%, *p* < 0.001). In addition, compared with participants having normal cognition, the number of participants with MCI was significantly higher among those diagnosed with type 2 diabetes (20.2% vs. 12.4%, *p* = 0.026) and those with an increased BMI (25.8 ± 3.9 vs. 24.8 ± 3.8 kg/m2, *p* = 0.007) **(**Table 1**)**.

The median intake in each dietary intake group (fresh red meat, processed meat, white meat, animal protein, fruits, vegetables, drinks with added sugar, refined carbohydrates, and unrefined carbohydrates) and the numbers of participants with normal cognition and MCI in each tertile are shown in Additional file 1: Table S1. In higher levels of fresh red meat and lower levels of drinks with added sugar intake, the number of participants with MCI and dementia was significantly lower than that of participants with normal cognition (*p* = 0.010 and *p* = 0.013).

### Association between BMI and risk of MCI and Dementia

The univariate multinomial logistic analysis showed that age of ≥ 60 years, education level of < 7 years, BMI of ≥ 25 kg/m^2^, and type 2 diabetes were potential factors associated with MCI, whereas the age of ≥ 60 years and education level of < 7 years were potential factors associated with dementia **(**Table 2 and Additional file 1: Table S3). Furthermore, after controlling for potential covariates (age, education level, and type 2 diabetes), a BMI of ≥ 25 kg/m^2^ (odds ratio, 1.91 [95% confidence interval, 1.12–3.26], *p* = 0.017) was also associated with a greater risk of MCI. In contrast, no significant association between BMI levels and the risk of dementia was found (Table 2). Additionally, in the multivariate multinomial logistic models, age of ≥ 60 years and an education level of < 7 years were independently associated with a greater risk of MCI (1.70 [1.02–2.84], *p* = 0.042 and 3.23 [2.07–5.02], *p* < 0.001, respectively) and dementia (5.26 [2.47–11.23], *p* < 0.001 and 4.36 [2.04–9.35], *p* < 0.001, respectively).

**Table 2:** Association of body mass index with mild cognitive impairment and dementia using multinomial logistic regression analysis

	**Univariate regression model**				**Multivariate regression model**^**a**^			
	**Risk of mild cognitive impairment **		**Risk of dementia**		**Risk of mild cognitive impairment **		**Risk of dementia**	
	**Odds ratio (95% ** **CI** **)**	***p*** **-value**	**Odds ratio (95% CI)**	***p*** **-value**	**Odds ratio (95% ** **CI** **)**	***p*** **-value**	**Odds ratio (95% CI)**	***p*** **-value**
Body mass index (kg/m^2^)								
18.5–22.9	Reference		Reference		Reference		Reference	
<18.5	1.63 (0.42–6.35)	0.480	2.72 (0.52–14.14)	0.234	2.04 (0.51–8.16)	0.311	2.33 (0.39–14.06)	0.357
23.0–24.9	1.49 (0.82–2.69)	0.188	0.90 (0.33–2.44)	0.833	1.47 (0.80–2.72)	0.216	0.97 (0.34–2.80)	0.952
≥25.0	2.04 (1.23–3.38)	**0.006**	1.06 (0.46–2.44)	0.886	1.91 (1.12–3.26)	**0.017**	1.14 (0.46–2.76)	0.783

CI, confidence interval

Boldface *p*-values are statistically significant.

– Results are not available due to low sample sizes of dementia event.

^a^Multivariable multinomial logistic regression model was adjusted for age, education level, and type 2 diabetes.

### Association between Dietary Intake and Risk of MCI and Dementia

The univariate multinomial logistic analysis showed that fresh red meat consumption was inversely associated with the risk of MCI (*p* for trend = 0.040) and dementia (*p* for trend = 0.005). Compared with the first tertile, fresh red meat consumption in the third tertile was also associated with a lower risk of MCI (*p* = 0.032) and dementia (*p* = 0.006). However, compared with the first tertile, the third tertile of drinks with added sugar was associated with a higher risk of MCI (*p* = 0.026) and dementia (*p* = 0.008). After controlling for age, education level, type 2 diabetes, and BMI, fresh red meat consumption was still inversely associated with the risk of MCI (*p* for trend = 0.037) and dementia (*p* for trend = 0.001) and, compared with the first tertile, fresh red meat consumption in the third tertile was associated with a lower risk of MCI (odds ratio, 0.57 [95% confidence interval, 0.34–0.96], *p* = 0.034) and dementia (0.25 [0.08–0.76], *p* = 0.015) (Table 3).

**Table 3:** Association of dietary intake with mild cognitive impairment and dementia using univariate and multivariate multinomial logistic regression analyses

**Dietary intake**	**Tertile**	**Univariate regression models**						**Multivariate regression models**^**a**^					
		**Risk of mild cognitive impairment**			**Risk of dementia**			**Risk of mild cognitive impairment**			**Risk of dementia**		
		**Odds ratio (95% CI)**	***p*** **-value**	***p*** ** for trend**	**Odds ratio (95% CI)**	***p*** **-value**	***p*** ** for trend**	**Odds ratio (95% CI)**	***p*** **-value**	***p*** ** for trend**	**Odds ratio (95% CI)**	***p*** **-value**	***p*** ** for trend**
Fresh red	T1	Reference		**0.040**	Reference		**0.005**	Reference		**0.037**	Reference		**0.011**
meat	T2	1.00 (0.63–1.58)	0.992		0.65 (0.29–1.44)	0.287		0.86 (0.53–1.39)	0.530		0.57 (0.24–1.35)	0.203	
	T3	0.58 (0.35–0.95)	**0.032**		0.22 (0.07–0.65)	**0.006**		0.57 (0.34–0.96)	**0.034**		0.25 (0.08–0.76)	**0.015**	
Processed	T1	Reference		0.110	Reference		0.867	Reference		0.238	Reference		0.870
meat	T2	0.70 (0.42–1.17)	0.173		0.59 (0.21–1.63)	0.308		0.72 (0.42–1.24)	0.242		0.49 (0.17–1.45)	0.198	
	T3	0.70 (0.44-–1.13)	0.142		0.98 (0.45–2.14)	0.963		0.76 (0.46–1.26)	0.290		1.20 (0.51–2.81)	0.679	
White meat	T1	Reference		0.288	Reference		0.865	Reference		0.227	Reference		0.589
	T2	0.80 (0.50–1.28)	0.352		0.58 (0.23–1.43)	0.233		0.78 (0.47–1.28)	0.316		0.55 (0.21–1.45)	0.230	
	T3	0.77 (0.48–1.25)	0.291		0.93 (0.42–2.07)	0.854		0.74 (0.45–1.21)	0.230		1.29 (0.54–3.10)	0.566	
Animal	T1	Reference		0.232	Reference		0.591	Reference		0.085	Reference		0.519
protein	T2	0.96 (0.60–1.53)	0.856		0.40 (0.15–1.06)	0.065		0.93 (0.57–1.52)	0.768		0.93 (0.57–1.52)	0.533	
	T3	0.74 (0.46–1.21)	0.228		0.82 (0.38–1.77)	0.608		0.64 (0.38–1.06)	0.083		0.64 (0.38–1.06)	0.130	
Fruits	T1	Reference		0.405	Reference		0.963	Reference		0.676	Reference		0.946
	T2	0.76 (0.47–1.22)	0.255		0.85 (0.36–2.03)	0.715		0.94 (0.56–1.56)	0.801		1.27 (0.50–3.24)	0.611	
	T3	0.82 (0.52–1.32)	0.416		0.98 (0.43–2.25)	0.962		0.90 (0.55–1.47)	0.677		1.03 (0.42–2.52)	0.942	
Vegetables	T1	Reference		0.396	Reference		0.452	Reference		0.399	Reference		0.371
	T2	0.81 (0.51–1.29)	0.376		0.94 (0.42–2.10)	0.886		0.87 (0.53–1.42)	0.575		1.19 (0.51–2.79)	0.686	
	T3	0.83 (0.51–1.35)	0.448		0.69 (0.28–1.73)	0.430		0.81 (0.49–1.35)	0.417		0.59 (0.22–1.58)	0.298	
Drinks with added sugar	T1	Reference		0.084	Reference		**0.005**	Reference		0.869	Reference		0.102
	T2	0.86 (0.52–1.44)	0.569		2.81 (0.89–8.90)	0.080		0.73 (0.43–1.24)	0.244		2.80 (0.88–8.90)	0.081	
	T3	1.72 (1.07–2.78)	**0.026**		4.53 (1.50–13.72)	**0.008**		1.02 (0.61–1.69)	0.945		2.60 (0.79–8.54)	0.115	
Refined	T1	Reference		0.647	Reference		0.567	Reference		0.217	Reference		0.746
carbohydrates	T2	1.30 (0.83–2.04)	0.250		1.53 (0.63–3.68)	0.347		1.10 (0.68–1.76)	0.703		1.46 (0.56–3.80)	0.435	
	T3	0.84 (0.49–1.44)	0.519		1.32 (0.54–3.21)	0.547		0.67 (0.38–1.20)	0.177		1.21 (0.46–3.19)	0.701	
Unrefined	T1	Reference		0.391	Reference		0.361	Reference		0.674	Reference		0.608
carbohydrates	T2	0.73 (0.45–1.18)	0.200		0.47 (0.15–1.50)	0.199		0.87 (0.53–1.45)	0.599		0.58 (0.71–1.93)	0.371	
	T3	0.83 (0.52–1.33)	0.440		2.20 (0.91–4.77)	0.470		0.91 (0.56–1.48)	0.698		2.07 (0.91–4.71)	0.082	

*CI* confidence interval

Boldface *p*-values are statistically significant.

^a^Each multivariable multinomial logistic regression model was adjusted for age, education level, type 2 diabetes, and body mass index.

## Discussion

This population-based longitudinal study revealed that the incidence rates of MCI and dementia were 42.5 and 11.2 per 1,000 person-years, respectively. A BMI of ≥ 25 kg/m^2^ was independently associated with a higher risk of MCI at five years. An age of ≥ 60 years and education level of < 7 years were independently also associated with a higher risk of MCI and dementia at five years. Furthermore, fresh red meat intake was inversely associated with MCI and dementia at five years.

This study showed that a BMI of ≥ 25 kg/m^2^ (obesity) was associated with a higher risk of MCI than was a normal BMI. A recent study of people aged 50 to 65 years also revealed that BMIs of < 18.5 and ≥ 25 kg/m^2^ were associated with a higher risk of MCI [39]. However, the present study showed no association between BMI and a higher risk of dementia. The association between the BMI and cognitive impairment remains controversial [14, 40–42]. People with obesity have an increased risk of insulin resistance and metabolic syndrome, which may account for the pathophysiology of cerebrovascular diseases and may progress to vascular dementia, the second most common cause of dementia. In addition, adipose cells produce inflammatory cytokines, leading to neurological damage and cognitive decline. Nonetheless, we found neither association between being underweight and MCI nor an association between low or high BMI and dementia in the present study. The reason for this may be that too few participants had a BMI of < 18.5 kg/m^2^. Also, the number of participants with dementia was small. Although the baseline characteristic of a previous diagnosis of stroke was not different between the groups, the participants who had dementia at the 5-year follow-up had no stroke diagnosis. Because of the retrospective nature of this study, the diagnosis of stroke was obtained either from the participants themselves or, if they had died, from the national record – extracted from their death certificate; no brain imaging was utilized.

The present study showed that the amount of fresh red meat consumption was inversely associated with the risk of MCI and dementia. A previous study also showed that a high intake of unprocessed meat was associated with a lower risk of dementia [26]. Fresh red meat is a highly valued source of cognition-related nutrients, including protein, iron, zinc, niacin, cobalamin, and riboflavin [43]. In older adults, adequate protein intake can reduce the risk of MCI and dementia [44]. Moreover, inadequate iron intake results in iron deficiency anemia, a reversible cause of the cognitive decline and intentional deficit. Chronic brain hypoxia related to anemia may contribute to a decline in cognitive function through an increasing accumulation of amyloid-β [45, 46]. The bioavailability of heme iron in fresh red meat is much higher than that of non-heme iron in plants [47]. Conversely, high iron intake can increase the risk of non-communicable diseases, such as type 2 diabetes and atherosclerosis. In addition, iron loading may result in brain iron deposition and neurodegeneration. The pathophysiology is related to oxidative stress and changes in the activity of transcription factors (nuclear factor κB and activator protein 1) [43]. The World Cancer Research Fund International recommended that red meat consumption should not exceed 500 g/week [48]; however, the lowest recommended amount of red meat consumption was not mentioned. In this study, the median intake of fresh red meat among the participants in the third tertile was 100 g/day. Accordingly, adequate fresh red meat, but not processed meat, may be a protective factor against cognitive impairment.

MCI and dementia are gradually increasing in prevalence and becoming a burden in aging populations. On the background of the controversy regarding the association of nutritional status and dietary intake with cognitive impairment, the present study has added more information on the risk factors for MCI and dementia, particularly in the Asian population. This longitudinal study was conducted in a well-designed cohort. The study participants were EGAT employees with a wide range of sociodemographic backgrounds. However, the current study had some limitations that should be considered. In terms of employment status, people with severe frailty, illness, and disability were not included. The participants’ BMI and dietary intake may have changed during the 5-year study period. Because dietary data were collected using a self-reported dietary frequency questionnaire, energy intake could not be accurately evaluated. Additionally, some potential factors, including lifestyle, physical activity, and body composition, were not analyzed because of unavailable data. Lastly, more than a quarter of participants lost to follow-up. Nevertheless, the baseline characteristics of participants between the analyzed data and lost follow-up groups were not significantly different. Further well-controlled, prospective studies with a large sample size are required to confirm the effects of BMI and meat consumption on cognitive impairment.

## Conclusion

Obesity was associated with a greater risk of 5-year MCI. Advanced age and low education levels were independently associated with a greater risk of 5-year MCI and dementia. In terms of dietary intake, low consumption of fresh red meat could be a risk factor for MCI and dementia in five years.

## Supplementary Information

**Additional file 1.**

## Data Availability

The datasets used and/or analyzed during the current study are available from the corresponding author on reasonable request.

## Abbreviations

ADL: Activities of daily living
BI: Barthel index
BMI: Body mass index
DASH: Dietary Approaches to Stop Hypertension
EGAT: Electricity Generating Authority of Thailand
FFQ: Food frequency questionnaire
IADL: Instrumental activities of daily living
K10: 10-item Kessler Psychological Distress Scale
L-IADL: Lawton instrumental activities of daily living
MCI: Mild cognitive impairment
MoCA: Montreal Cognitive Assessment
