# Supplementary material for: Association of body mass index and dietary intake with mild cognitive impairment and dementia: a retrospective cohort study
Source: BMC Geriatr. 2023 Jan 3;23:3. doi: 10.1186/s12877-022-03700-5 (PMC9808972; doi:10.1186/s12877-022-03700-5)
Supplement: Supplementary file 1 — Additional file 1. [file 12877_2022_3700_MOESM1_ESM.docx]

**Table S1. Median intake and numbers of participants in each tertile of dietary intake stratified by cognitive status at 5-year follow-up (normal cognition, MCI, and dementia)**

| **Dietary intake** | **Tertile** | **Median**  **(min–max)** | **Cognitive status at 5-year follow-up** | | | | | |
| --- | --- | --- | --- | --- | --- | --- | --- | --- |
|  |  |  | **Normal**  **N (%)** | **MCI**  **N (%)** | ***p*-value**  **Normal**  **VS.**  **MCI** | **Dementia**  **N (%)** | ***p*-value**  **Normal**  **VS.**  **Dementia** | ***p*-value** |
|  |  |  |  |  |  |  |  |  |
| Fresh red meat | T1 | 14.3 (0.0–16.9) | 165 (37.2) | 56 (43.4) | **0.026** | 50 (58.8) | **0.013** | **0.010** |
| (g/day) | T2 | 28.6 (17.0–43.0) | 127 (28.6) | 43 (33.3) |  | 10 (29.4) |  |  |
|  | T3 | 100.0 (43.1–300.0) | 152 (34.2) | 30 (23.3) |  | 4 (11.8) |  |  |
| Processed meat | T1 | 0.0 (0.0–0.0) | 212 (47.7) | 73 (56.6) | 0.209 | 18 (52.9) | 0.569 | 0.396 |
| (g/day) | T2 | 3.3 (0.1–10.0) | 100 (22.5) | 24 (18.6) |  | 5 (14.7) |  |  |
|  | T3 | 14.3 (10.1–100.0) | 132 (29.7) | 32 (24.8) |  | 11 (32.4) |  |  |
| White meat | T1 | 31.4 (0.0–49.9) | 141 (31.8) | 48 (37.2) | 0.507 | 13 (38.2) | 0.449 | 0.592 |
| (g/day) | T2 | 71.4 (50.0–100.0) | 151 (34.0) | 41 (31.8) |  | 8 (23.5) |  |  |
|  | T3 | 157.1 (100.1–757.0) | 152 (34.2) | 40 (31.0) |  | 13 (38.2) |  |  |
| Animal protein | T1 | 84.3 (0.0–120.9) | 145 (32.7) | 47 (36.4) | 0.437 | 15 (44.1) | 0.165 | 0.256 |
| (g/day) | T2 | 157.1 (121.0–199.9) | 145 (32.7) | 45 (34.9) |  | 6 (17.6) |  |  |
|  | T3 | 303.3 (200.0–886.0) | 154 (34.7) | 37 (28.7) |  | 13 (38.2) |  |  |
| Fruits | T1 | 28.6 (0.0–46.9) | 148 (33.3) | 50 (38.8) | 0.494 | 12 (35.3) | 0.926 | 0.827 |
| (g/day) | T2 | 100.0 (47.0–102.9) | 145 (32.7) | 37 (28.7) |  | 10 (29.4) |  |  |
|  | T3 | 200.0 (103.0–1000.0) | 151 (34.0) | 42 (32.6) |  | 12 (35.3) |  |  |
| Vegetables | T1 | 28.6 (0.000–46.9) | 188 (42.3) | 61 (47.3) | 0.606 | 16 (47.1) | 0.723 | 0.817 |
| (g/day) | T2 | 85.7 (47.0–100.0) | 137 (30.9) | 36 (27.9) |  | 11 (32.4) |  |  |
|  | T3 | 164.3 (100.1–714.0) | 119 (26.8) | 32 (24.8) |  | 7 (20.6) |  |  |
| Drinks with added sugar | T1 | 21.5 (0.0–64.4) | 144 (32.4) | 56 (43.4) | 0.061 | 4 (11.8) | **0.015** | **0.013** |
| (mL/day) | T2 | 150.0 (64.5–214.4) | 155 (34.9) | 35 (27.1) |  | 12 (35.3) |  |  |
|  | T3 | 347.9 (214.5–1098.0) | 145 (32.7) | 38 (29.5) |  | 18 (52.9) |  |  |
| Refined carbohydrates | T1 | 110.0 (7.0–174.4) | 150 (33.8) | 41 (31.8) | 0.253 | 9 (26.5) | 0.638 | 0.487 |
| (g/day) | T2 | 249.5 (174.5–321.9) | 142 (32.0) | 51 (39.5) |  | 13 (38.2) |  |  |
|  | T3 | 400.0 (323.0–900.0) | 152 (34.2) | 37 (28.7) |  | 12 (35.3) |  |  |
| Unrefined carbohydrates | T1 | 0.0 (0.0–14.2) | 173 (39.0) | 58 (45.0) | 0.421 | 11 (32.4) | 0.333 | 0.320 |
| (g/day) | T2 | 42.9 (14.3–100.0) | 135 (30.4) | 33 (25.6) |  | 4 (11.8) |  |  |
|  | T3 | 200.0 (100.1–714.0) | 136 (30.6) | 38 (29.5) |  | 19 (55.9) |  |  |

MCI, mild cognitive impairment; min, minimum; max, maximum

**Table S2. Association of potential factors with mild cognitive impairment and dementia using univariate multinomial logistic regression analysis**

| **Baseline characteristics** | **Risk of mild cognitive impairment** | | **Risk of dementia** | |
| --- | --- | --- | --- | --- |
|  | **Odds ratio**  **(95% confidence interval)** | ***p*-value** | **Odds ratio**  **(95% confidence interval)** | ***p*-value** |
| Age (≥60 years) | 1.74 (1.07–2.82) | **0.026** | 5.09 (2.47-10.48) | **<0.001** |
| Sex (male) | 1.41 (0.90–2.21) | 0.131 | 0.75 (0.37-1.54) | 0.437 |
| Body mass index (kg/m^2^) |  |  |  |  |
| 18.5–22.9 (reference) |  |  |  |  |
| <18.5 | 1.63 (0.42–6.35) | 0.480 | 2.72 (0.52-14.14) | 0.234 |
| 23.0–24.9 | 1.49 (0.82–2.69) | 0.188 | 0.90 (0.33-2.44) | 0.833 |
| ≥25.0 | 2.04 (1.23–3.38) | **0.006** | 1.06 (0.46-2.44) | 0.886 |
| Education level (<7 years) | 3.32 (2.16–5.12) | **<0.001** | 4.01 (1.95-8.26) | **<0.001** |
| Underlying diseases |  |  |  |  |
| Dyslipidemia | 1.07 (0.97–1.18) | 0.211 | 1.03 (0.87-1.23) | 0.742 |
| Hypertension | 1.25 (0.84–1.85) | 0.276 | 1.28 (0.63-2.60) | 0.492 |
| Type 2 diabetes | 1.79 (1.07–2.99) | **0.027** | 1.22 (0.45-3.28) | 0.695 |
| Ischemic heart disease | 0.97 (0.72–1.29) | 0.809 | 0.82 (0.56-1.20) | 0.302 |
| Thyroid disorder | 1.40 (0.84–2.34) | 0.195 | 0.83 (0.57-1.22) | 0.351 |
| Stroke | 0.73 (0.49–1.09) | 0.128 | - | - |
| Smoking | 0.97 (0.84–1.02) | 0.129 | 1.08 (0.89-1.31) | 0.438 |
| Alcohol drinking | 1.01 (0.90–1.13) | 0.852 | 1.06 (0.88-1.29) | 0.549 |

Boldface *p*-values are statistically significant.

– Results are not available due to low sample sizes of dementia event.

**Table S3. Baseline characteristics of participants with analyzed data and loss follow-up**

| **Baseline characteristics** | **Analyzed data** | **Loss follow-up** | ***p*-value** |
| --- | --- | --- | --- |
|  | **N = 607 (73.9%)** | **N = 214 (26.1%)** |  |
| Age, years | 55.9 ± 4.4 | 56.3 ± 4.0 | 0.187 |
| Age of ≥60 years | 112 (18.5) | 30 (14.0) | 0.140 |
| Sex, male | 421 (69.4) | 163 (76.2) | 0.059 |
| Body mass index, kg/m^2^ | 25.0 ± 3.9 | 25.2 ± 3.7 | 0.653 |
| Body mass index, kg/m^2^ |  |  | 0.910 |
| <18.5 | 15 (2.5) | 5 (2.3) |  |
| 18.5–22.9 | 171 (28.2) | 55 (25.7) |  |
| 23.0–24.9 | 142 (23.4) | 53 (24.8) |  |
| ≥25.0 | 279 (46.0) | 101 (47.2) |  |
| Education level of <7 years | 139 (22.9) | 57 (26.6) | 0.270 |
| Underlying diseases |  |  |  |
| Dyslipidemia | 330 (54.4) | 102 (47.7) | 0.091 |
| Hypertension | 329 (54.2) | 132 (61.7) | 0.058 |
| Type 2 diabetes | 86 (14.2) | 40 (18.7) | 0.114 |
| Ischemic heart disease | 18 (3.0) | 5 (2.3) | 0.632 |
| Thyroid disorder | 16 (2.6) | 5 (2.3) | 0.811 |
| Stroke | 6 (1.0) | 4 (1.9) | 0.313 |
| Smoking | 226 (37.2) | 91(42.5) | 0.171 |
| Alcohol drinking | 454 (74.8) | 168 (78.5) | 0.276 |

Data are presented as mean ± standard deviation or n (%).
